# Microbiome-Gut-Brain Axis and Toll-Like Receptors in Parkinson’s Disease

**DOI:** 10.3390/ijms19061689

**Published:** 2018-06-06

**Authors:** Valentina Caputi, Maria Cecilia Giron

**Affiliations:** 1 Pharmacology Building, Department of Pharmaceutical and Pharmacological Sciences, University of Padova, 35131 Padova, Italy; valentina.caputi@ucc.ie; 2 APC Microbiome Ireland, University College Cork, T12YT20 Cork, Ireland

**Keywords:** enteric microbiota, brain-gut axis, Parkinson’s disease, toll-like receptors, innate immunity, central nervous system, enteric nervous system, gastrointestinal dysfunctions, probiotics, pharmacological treatment, α-synuclein, gut dysbiosis, neurons, microglia, glial cells, intestinal barrier permeability

## Abstract

Parkinson’s disease (PD) is a progressively debilitating neurodegenerative disease characterized by α-synucleinopathy, which involves all districts of the brain-gut axis, including the central, autonomic and enteric nervous systems. The highly bidirectional communication between the brain and the gut is markedly influenced by the microbiome through integrated immunological, neuroendocrine and neurological processes. The gut microbiota and its relevant metabolites interact with the host via a series of biochemical and functional inputs, thereby affecting host homeostasis and health. Indeed, a dysregulated microbiota-gut-brain axis in PD might lie at the basis of gastrointestinal dysfunctions which predominantly emerge many years prior to the diagnosis, corroborating the theory that the pathological process is spread from the gut to the brain. Toll-like receptors (TLRs) play a crucial role in innate immunity by recognizing conserved motifs primarily found in microorganisms and a dysregulation in their signaling may be implicated in α-synucleinopathy, such as PD. An overstimulation of the innate immune system due to gut dysbiosis and/or small intestinal bacterial overgrowth, together with higher intestinal barrier permeability, may provoke local and systemic inflammation as well as enteric neuroglial activation, ultimately triggering the development of alpha-synuclein pathology. In this review, we provide the current knowledge regarding the relationship between the microbiota-gut–brain axis and TLRs in PD. A better understanding of the dialogue sustained by the microbiota-gut-brain axis and innate immunity via TLR signaling should bring interesting insights in the pathophysiology of PD and provide novel dietary and/or therapeutic measures aimed at shaping the gut microbiota composition, improving the intestinal epithelial barrier function and balancing the innate immune response in PD patients, in order to influence the early phases of the following neurodegenerative cascade.

## 1. Introduction

Parkinson’s disease (PD) is acknowledged as the second most common neurodegenerative disorder, estimated to affect 1–2 per 1000 of the population worldwide [1]. About seven to ten million people in the world suffer from PD [2]. This figure is expected to double in the near future, circa 2030, due to aging of the population [3]. The etiology of PD still remains unclear. However, the slow progression of the disease evolves years before the diagnosis is ascertained, involving various neuroanatomical areas, arising from an assortment of genetic and environmental factors, and exhibiting a large array of debilitating symptoms. From a histopathological perspective, PD is hallmarked by a distinctive depauperation of dopaminergic neurons in the substantia nigra pars compacta (SNc) with consequent dopamine deficiency within the striatum and by the manifestation of intracellular eosinophilic inclusions, the so called Lewy bodies and Lewy neurites in the remaining neurons. Lewy pathology is characterized by intracellular insoluble aggregates of misfolded α-synuclein and implicates not only the brain but is also widespread in the spinal cord and peripheral nervous system, including sympathetic ganglia, enteric nervous system (ENS), salivary glands, adrenal medulla, vagus nerve, cutaneous nerves and the sciatic nerve [4,5,6]. The progressive dopamine deficit in the basal ganglia determines the characteristic parkinsonian triad of motor symptoms—rigidity, bradykinesia and tremor. However, other significant non-motor symptoms have been receiving increasing attention for the more negative impact in the quality of life of PD patients in comparison to motor symptoms. These non-motor symptoms involve neuropsychiatric disorders (e.g., cognitive impairment, depression, apathy, psychosis), sleep disturbances, sensory alterations (pain, olfactory impairment), and the common gastrointestinal (GI) dysfunction reported by more than 80% of PD patients [7]. The first-line intervention in PD management is the administration of dopamine modulators even if they can exert serious side effects, produce limited benefits on alleviating non-motor disturbances and often fail to be effective in the later stages of PD [6,7,8].

In the last decade, emerging evidence has revealed the presence of an intense dialogue between the brain and the GI system, the so-called brain-gut axis. Disruption of this complex relationship has been shown to be associated to the pathogenesis of several disorders, ranging from irritable bowel syndrome (IBS), liver disease and chronic abdominal pain syndromes, to depression, anxiety, autism spectrum disorders, dementia and PD. The brain-gut (or gut-brain axis) crosstalk can occur in a bidirectional fashion: firstly, through a gut quiver on central nervous system (CNS) activities (e.g., changes in mood, cognition or perception due to functional GI disorders [9,10], or subsequent to the release of gut hormones [11], or following serious GI inflammatory diseases such as acute pancreatitis [12,13,14]); secondly, through a central quiver on gut activities (e.g., stress-induced GI dysfunction [15]). Indeed, a common alignment of both brain and gut can be identified in neurobiological disorders (e.g., neurodegeneration or gliosis) [16].

Traditionally, the brain-gut axis was viewed as a flux of information, mediated by neurohormones and inflammatory factors, travelling between the central, autonomic and enteric nervous systems (CNS, ANS and ENS, respectively) with the concurrent participation of the neuroendocrine and neuroimmune systems.

Several studies have focused on the role of the CNS in modulating the intestinal inflammation through both parts of the ANS, the sympathetic and parasympathetic nervous system [17,18]. The inflammatory state of peripheral tissues is conveyed to the brain through afferent nerves which in turn suppress cytokine production, improve intestinal barrier integrity and limit gut inflammation [18]. Recent studies performed in animal models of acute and chronic pancreatitis have shown that the stimulation of primary afferent capsaicin-sensitive neurons or treatment with peptides (e.g., calcitonin gene-related peptide) before the exposure to harmful factors, can activate an adaptive mechanism called “preconditioning” which is able to reduce pancreatitis development [19,20,21]. Sensory neurons are involved in gastroprotection and regulation of visceral blood flow and their stimulation by capsaicin can potentially inhibit the progression of inflammation, by improving the endogenous release of nitric oxide (NO) and thus the pancreatic blood flow [22,23,24]. In recent years, several preclinical studies highlighted that certain psychoactive molecules can modulate the endocannabinoid system in the gut and possibly impact the pathogenesis of inflammatory bowel disease, as well as its extra intestinal manifestations such as pancreatitis [25,26]. It has been demonstrated by Warzecha et al. [25] that anandamide reduces mucosal oxidative stress, inhibits the inflammatory process and preserves the integrity of gastric mucosa in stress-induced gastric ulcers [25]. These effects are partly mediated by capsaicin-sensitive sensory nerves [25] and, in the case of acute pancreatitis, the protective action of anandamide depends on the phase of the inflammation [27,28]. Thus, the modulation of the endocannabinoid system may be useful to treat gut-brain motor dysfunction in PD.

Changes in the ANS occurred in conjunction with intestinal inflammation, however, disorders such as IBS are also linked to inflammatory abnormalities of the ENS [17]. The ENS is the largest nervous system outside the CNS, which autonomously regulates numerous functions of the GI tract, either independently through neuro-glial circuits in the myenteric and submucosal plexus, or by input of sympathetic and parasympathetic pathways to/from the brain. The enteric neurons and glial cells form a vast communication network in close relationship with the gut microbiota and thus the ENS can easily be affected by microbiome alterations and be involved in GI disorders as well as in neurodegenerative diseases. Therefore, the ENS could represent an entry point for pathogens or—conversely—for therapeutic interventions based on diet and/or commensal microbes-derived molecules [29].

Lately, it is becoming increasingly clear that a third player, such as the gut microbiota, can significantly influence the gut-brain crosstalk, having a marked impact on digestive processes, immune responses, emotional status, perception and cognitive functions [16]. The microbiota-gut-brain axis has attracted much attention regarding the pathogenesis of PD, in which GI dysfunction appears about twenty years before motor impairments. Although PD patients manifest both gut dysmotility and altered microbial composition, it is still unclear which condition comes first and what role the gut and the gut microbiota have in PD progression.

In addition to maintaining gut homeostasis and several essential host physiological functions, the gut microbiota is a producer of an assortment of Toll-like receptor (TLR) ligands, which can exert proinflammatory effects under certain conditions. Despite microbial-derived components being potent TLR ligands, the gut has a high tolerance to TLR ligands because epithelial cells express minimal TLRs under physiological conditions [30]. In contrast, altered gut microbiota and disrupted gut epithelial barrier activate TLRs which in turn trigger downstream signaling pathways, promoting inflammation and oxidative stress in both the gut and brain of PD patients. Thus, gut microbiota and TLRs could represent potential targets for PD treatment.

The exact mechanisms by which gut microbiota contribute to PD are still poorly understood, despite the role of gut microbiota in the development of PD being well documented. Here, we first describe the functional aspects of gut microbiota observed in PD. Then, we review the role of TLRs associated with PD and their potential as a new target of dietary and/or therapeutic interventions.

## 2. Microbiota-Gut-Brain Axis and Host Health

Over the last decade, an increasing amount of literature has focused on the codevelopment of the gut microbiota with the human host since birth and on their mutual shaping clearly relying on the host genome, nutrition and lifestyle [31]. While the association of neuropsychiatric disorders with GI disturbances dates back to Hippocrates, a clear demonstration of the essential cooperation between brain and microbes was first described by the impressive amelioration of symptoms in patients affected by hepatic encephalopathy following treatment with nonadsorbable oral antibiotics [32]. The gut microbiota is now being referred to as a new organ or an emergent system, which comprises a number of microorganisms (bacteria, archaea, fungi, and viruses) comparable to the number of cells residing in the human body [33]. In particular, the enteric microbiota, distributed along the human GI tract, displays similar results in terms of relative abundance and distribution between healthy adults, although the microbe profile is quite stable and unique for each individual, and can be considered a personal microbic fingerprint or enterotype [34]. *Firmicutes* and *Bacteriodetes* are the most dominant phyla (about 51% and 48%, respectively), with *Actinobacteria* (including the Bifidobacteria genera), *Cyanobacteria*, *Lentisphaerae*, *Fusobacteria*, *Spirochaetes*, *Proteobacteria*, and *Verrucomicrobia* phyla existing in relatively low abundance [35]. Although a relative consistency in microbial composition in healthy people is usually maintained over time, small daily variations are found in each individual unless exposed to disrupting agents or conditions, such as antibiotics, colonization by foreign commensal microbes, marked changes in diet or lifestyle, or infectious or noninfectious disease [36,37,38]. The magnitude and the length of the disruption might affect the capability of microbiota to recover and return to the original composition once the dysfunction is resolved, however repeated harmful stimuli will weaken its recovery with potential downstream outcomes on host physiology [38].

Aging is a critical window for not only the gut and brain function but also for the composition of enteric microbiota that in turn may have serious consequences on health integrity in these latter stages of life [38].

The bidirectional dialogue between the gut and the brain involves different mechanisms, including the enteric and central neural network, neuroendocrine-hypothalamic-pituitary-adrenal axis, immune system, several neurotransmitters and neural regulators directly produced by gut bacteria, and barrier paths such as intestinal mucosal barrier and blood-brain barrier. The enteric microbiota is implicated in the upregulation of the local and systemic inflammatory response induced by lipopolysaccharides (LPS) derived from pathogens and the related production of proinflammatory mediators. The dysregulation of immune response to environmental and/or microbial agents is associated with the onset of inflammatory bowel disease in genetically susceptible individuals [39]. On the other hand, exposition to low amounts of LPS in early life can affect the ability of the immune cells to produce the cytokines, increasing the resistance of the organism to systemic diseases such as pancreatic inflammation [40].

Gut dysbiosis and/or small intestinal overgrowth (SIBO) increase intestinal permeability and bacterial translocation, determining an immune system’s overresponse and consequent systemic and/or central nervous system (CNS) inflammation. Enteric bacterial cells possess the capacity to produce numerous neuroactive molecules, such as serotonin, catecholamines, glutamate, γ-amminobutyric acid (GABA) and short-chain-fatty acids (SCFAs) [41,42,43]. It has been proposed that the variety of neurotransmitters, neuromodulators and neurohormones produced by microorganisms are the “words” of a common language that enables a sophisticated synergic communication [44]. However, considering the extreme complexity of this communication network, it remains to be determined whether microbial neurochemicals are generated at an adequate level in respect to host production to exert any kind of effect, or can be delivered to central neurocircuits through systemic circulation [41,42]. Although some reports indicate the ability of bacteria to modulate the level of neurotransmitters through TLRs and heat-shock proteins [45], this form of interaction may occur directly via neuroactive molecules.

Most of the functions of the GI tract are regulated by enteric neurons, hormones produced by enteroendocrine cells, and cytokines synthesized in somatic cells [46]. Of note, more than 90% of all serotonin in the body is synthesized in the gut by the enterochromaffin cells, an enteroendocrine cell subtype, known to be involved in controlling gut motility, emesis, visceral hypersensitivity and secretion [47]. Most enteric serotonin is tuned by gut microbiome whereas the circulating serotonin is generally metabolized by the liver [48]. This neuroamine regulates a variety of physiological processes at the periphery but, even if it cannot cross the brain blood barrier (BBB), can affect central neurocircuits by interfering with vagal nerve activity and BBB permeability, thus indirectly influencing brain functions [49]. Therefore, although the brain and the gut are the two major sources of serotonin synthesis, physically separated by the BBB, from a biological point of view these pathways are potentially not always distinct. In this respect, enteric bacteria can also produce neurotoxic molecules (e.g., d-lactic, ammonia) and neurotoxins, (e.g., those produced by *Clostridium perfringens*, *Clostridium botulinum*, *Clostridium butyricum*, *Clostridium baratii*, among others) and reach CNS being transported via systemic circulation or through extrinsic afferent nerve fibers (i.e., vagal nerve projections from the gut to the brainstem and spinal nerve projections from the gut to the spinal cord), provoking neuronal injury [50,51]. In turn, the CNS can control the enteric microbiota through adrenergic neurotransmission, primarily modulating intestinal motility and neuroimmune crosstalk [52].

Most of the effects mediated by the gut microbiota or potential food-based therapeutic interventions (e.g., probiotics, prebiotics, synbiotics) on CNS functionality have been shown to be exerted through the modulation of vagal neurotransmission [53]. Likewise, microbial colonization of the gut after birth and during infancy is markedly involved in the postnatal development and maturation of the host nervous, immune, and endocrine systems. Altogether these pieces of evidence highlight the key role of microbiota-gut-brain axis in ensuring and protecting host health and its involvement in a plethora of diseases ranging from stress-induced disorders to neurologic and psychiatric disorders [53].

## 3. The Gastrointestinal System in Parkinson’s Disease

GI dysfunctions are generally experienced by most patients with PD and usually include hypersalivation, dysphagia, delayed gastric emptying, nausea, constipation and altered bowel habits. Disturbances in oral and pharyngeal swallowing have been shown in other neurological diseases, such as amyotrophic lateral sclerosis, and manometric endoscopy of the upper GI tract was a useful procedure for the assessment of the severity of deglutition disorders among these patients [54].

A higher prevalence of peptic ulcer and *Helicobacter pylory* (Hp) infection has been revealed in PD patients. Hp infection is considered to be the most important factor responsible for the development of gastroduodenal diseases, including active chronic gastritis, peptic ulcer disease and gastric adenocarcinoma [55]. However, there is increasing evidence that Hp gastric infections are potentially associated with several systemic extra-GI diseases such as cardiovascular, immunological and inflammatory disorders (e.g., acute pancreatitis) [55].

Constipation is a major enteric dysfunction of PD and predates motor symptoms years before the manifestation of the disease, making it one of the earliest biomarkers of the pathological process that will ultimately emerge into PD [6]. The anomalies in gut functionality in PD may result from both central and peripheral altered neurotransmission pathways. Even if until now the findings of an impaired enteric neurotransmission are contradictory [56], a pathological hallmark of PD is the distribution of α-synuclein pathology in olfactory bulbs and in both submucosal and mucosal plexuses of gut ENS from esophagus to the rectal end. Braak et al. [57] have proposed that α-synuclein deposition might begin in the olfactory bulbs or/and in the ENS by an unknown environmental toxin and/or microbial pathogen and then proceed towards SNc and further sites in the CNS. Full truncal vagotomy in PD patients resulted in decreased risk of PD progression compared to partial or no vagotomy, suggesting an involvement of the vagus nerve as a conduit for the spreading of Lewy bodies from ENS to CNS [58]. Moreover, it has been shown that toxins like pesticides (e.g., rotenone), orally administered to mice, replicate the staging of PD-like pathology from ENS to CNS [35]. In this preclinical model, the resection of sympathetic and parasympathetic nerves (i.e., hemivagotomy and partial sympathectomy) prior to the oral exposure to rotenone prevented the spreading of PD-like pathology from ENS to the previously connected central neurocircuits and delay the manifestation of gut motor symptoms [59]. Considering that gut mucosa is in contact with environmental factors, these findings support the concept that external triggers, including diet-derived molecules, toxins or microbes, might have a major role in eliciting and spreading PD pathology, potentially in a milieu of genetic vulnerability.

However, recent studies have suggested that the initiation of α-synuclein pathology in the GI tract does not necessarily need a pathogenic or environmental trigger since it can be instigated by enteric microbiota [60,61].

## 4. The Gut Microbiota in Parkinson’s Disease

Gut constipation, SIBO, increased intestinal permeability of the mucosal barrier and GI inflammation are all symptoms closely interrelated to gut microbiota composition and microbial-derived metabolites. In the early stages of PD, higher intestinal permeability, a condition known as leaky gut syndrome, has been observed to be associated with enteric α-synuclein pathology [60]. Increased intestinal permeability, due to a defective gut barrier function, facilitates the translocation of microorganisms (e.g., bacteria) and microbial products (e.g., LPS), which, in turn, might initiate inflammation and oxidative stress, thereby leading to α-synucleinopathy in the ENS [60]. Moreover, higher levels of gut-derived LPS can disrupt the integrity of the BBB, promoting neuroinflammation and damage in SNc [62].

Current evidence from different clinical studies has revealed that gut microbiota composition is altered in PD patients and related to the clinical phenotype of the disease. Analysis of fecal samples of PD subjects disclosed higher levels of *Enterobacteriaceae*, which were positively associated with the degree of gait and postural instability [63], as well as a reduced abundance of the *Prevotellaceae* bacterial family. *Prevotellaceae* are commensals bacteria, implicated in the production of mucin in the gut mucosal layer, in the synthesis of neuroactive SCFAs (e.g., acetate, propionate, and butyrate) through fiber fermentation, and in the release of thiamine and folate. Hence, the decreased levels of *Prevotellaceae* could be related to reduced mucin synthesis and increased intestinal permeability, with consequent systemic exposure to bacterial antigens and endotoxins [60], and potential development of α-synucleinopathy by the disruption of clearance mechanisms of the intra- and extraneuronal protein by SCFA-dependent modulation of gene expression [64].

Furthermore, the lower abundance of *Prevotellaceae* associated with an increase of *Lactobacillaceae* has been linked to a reduction in the levels of gut hormone ghrelin, known to be involved in ensuring physiological nigrostriatal dopamine activity [65]. Indeed, in PD patients an impaired postprandial response to ghrelin has been reported [66].

A subsequent study demonstrated changes in both mucosal and fecal microbial compositions of PD, such as a higher abundance in the putative, proinflammatory *Proteobacteria* of the genus *Ralstonia* and a reduced level of bacteria from the genera *Blautia*, *Coprococcus*, and *Roseburia*, involved in producing butyrate, a SCFA associated with anti-inflammatory properties [61]. Furthermore, decreased concentrations in SCFA have also been evidenced in PD patients and could be related to the observed impaired peristaltic regulation by the ENS and consequent gut dysmotility. At a genomic level, PD microbiota owns more genes implicated in LPS biosynthesis and type III bacterial secretion systems and a lower number of genes related to metabolism [61]. Although the role of gut microbiota in PD is still at its infancy, a recent experimental work demonstrated that fecal microbiota transplant from PD-donor accelerated the disease in genetically predisposed animals [67]. These findings advocate that PD-associated factors in the host alter enteric microbiome, which in turn worsens PD pathology and symptoms. Indeed, the enteric microbiota is implicated in the regulation of the local and systemic inflammatory response induced by LPS and the production of proinflammatory mediators.

## 5. Toll-Like Receptors

Being the first line of defense against invading microbes, the innate immune system is vital in early recognition of infection [68]. Innate immunity senses the presence of microorganisms through a number of patter-recognition receptors (PRRs) [69], which specifically recognize evolutionarily conserved molecular structures, denominated pathogen-associated molecular patterns (PAMPs), widely expressed by a variety of infectious microbes, which are essential for their own survival [70]. Among the different PRRs, situated in both extracellular and intracellular milieu, TLRs are the most interesting family of mammalian receptors, orthologs of the Toll receptors discovered in Drosophila Melanogaster in 1988 [71]. Until now, thirteen TLRs have been identified in mammals, of which a total of ten have been characterized in a variety of human tissue and cells, not only belonging to the immune system and epithelial tissue but also to neurons and glial cells of both the peripheral nervous system (PNS) and the CNS. The distribution of these immune sensors in the nervous system highlights the ability of neural cells to mediate immune responses as well as their vital dependency for development and homeostasis on TLRs signaling [72,73,74,75,76]. Indeed, TLRs are not only engaged by PAMPs but also by a set of endogenous molecules generated during tissue damage (damage-associated molecular patterns, DAMPs), activating firstly an acute immune response and, secondarily, tuning the subsequent adaptive immune reaction. TLRs are further classified into subfamilies according to the types of PAMPs or DAMPs that they recognize. The plasma membrane bound TLRs interact with molecules composing the bacterial cell walls and membranes, such as lipopeptides, peptidoglycans and glycolipids (TLR1/TLR2, TLR2/TLR6, TLR2/TLR10), fibronectin, lipopolysaccharides, and heat shock proteins (TLR4), and flagellin (TLR5). On the contrary, the intracellular TLRs, expressed in cell endosomes, detect microbial nucleic acids, specifically viral double-strand RNAs (TLR3), single-strand RNAs (TLR7 and TLR8), unmethylated CpG DNA (TLR9) [77]. TLR engagement activates distinctive adaptor proteins, such as Myeloid differentiation primary response gene 88 (MyD88) or the TIR- domain-containing adapter-inducing interferon-β (TRIF) pathway, to deliver signals to several downstream pathways, comprising the nuclear factor-kappa B (NF-κB), mitogen-activated protein kinases (MAPK), and/or interferon-regulatory factor (IRF) signaling pathways, which are pivotal for the expression of a variety of gene products involved in inflammatory responses [77,78].

More and more studies have focused their attention on the role that TLRs may play in neurodegeneration, considering that they are extensively expressed in immune and non-immune cells and their expression can change not only during microbial infections but also in the presence of sterile inflammation when the pathogens are absent.

## 6. TLRs in Parkinson’s Disease

Even if the exact etiology of sporadic PD is still to be found, increasing evidence suggest that misfolded α-synuclein activates microglial cells in the SNc, promoting inflammation and oxidative stress, leading to neurodegeneration. Extracellular misfolded, fibrillar α-synuclein, released from neural cells or oligodendrocytes is recognized as PAMP or DAMP by microglial TLR2 (as heterodimer with TLR1), which in turn activates downstream pathways involving MyD88 and NF-κB, triggering the production of TNF and IL-1β [79,80] and increasing selective expression of TLRs in a localization- and time-dependent manner [81]. In in vitro studies TLR4 appears also to interact with α-synuclein, triggering microglial responses, including α-synuclein uptake, proinflammatory cytokine release, and oxidative stress promotion [82]. These findings were corroborated in an MPTP (1-methyl-4-phenyl-1,2,3,6-tetrahydropyridin)–induced murine model of PD where the genetic absence of TLR4 signaling protected the mice from neurodegeneration, highlighting the primary role of TLR4 in PD development [83]. Recent evidence in experimental models suggests a potential involvement of the NLRP3 inflammasome, which can be modulated by TLRs signaling. Reduced caspase-1 activation and IL-1β release together with decreased loss of dopaminergic neurons in SNc was found in NLRP3-deficient mice following MPTP insult [84]. Fibrillar α-synuclein has been reported to engage TLR2 on human monocytes to prime the NLRP3 inflammasome [85]. Intriguingly, NLRP3 inflammasome inhibition was shown to be mediated by dopamine itself, although it is still unclear if involvement of the inflammasome is antecedent to the degeneration of dopaminergic neurons or is the result of neural depauperation [86]. The most convincing evidence for a role of TLRs in PD has been found so far for TLR2 and TLR4. The contribution of TLR2 and TLR4 in PD might be a double-edged sword: their activation in microglia can trigger neurotoxicity but on the other hand they might be essential for clearing misfolded α-synuclein, hence being neuroprotective [87].

There is an increasing recognition of the involvement of TLRs in neuronal degeneration based on the following pieces of evidence: (i) relevant cells of the nervous system express TLRs; (ii) TLRs are activated by α-synuclein; (iii) their activation induces an inflammatory response that precedes neuronal loss; (iv) halting TLRs engagement delays PD progression [88].

## 7. Microbiota-Gut-Brain-Axis and Toll-Like Receptor Signaling: Potential Implications in Parkinson’s Disease

Aging determines alterations in responsivity of microglia, which may acquire a hyperreactive phenotype (e.g., increased release of proinflammatory mediators and overexpression of cell surface receptors) or an impaired condition with defective phagocytosis and clearance of misfolded protein aggregates [89]. The acquired activated state of microglia in response to accumulation of abnormally folded proteins and neurodegeneration is a process referred to as microglial priming, similar to the newly emerging theory of “trained immunity” or “innate immune memory”, consisting in the epigenetic and metabolic reprogramming of peripheral innate immune cells following an initial insult. Indeed, macrophages derived from human hematopoietic stem and progenitor cells challenged with a TLR2 agonist during their differentiation and then inoculated in irradiated mice evidenced a tolerant phenotype by releasing a reduced amount of inflammatory mediators and reactive oxygen species in response to the inflammatory stimulus [90]. A growing body of evidence suggests that microglial priming could be induced by epigenetic reprogramming as shown in trained immune cells [86]. Intestinal barrier function is impaired in patients with PD placing them at higher risk to be exposed to microbial products [91], therefore the translocation of bacteria or bacterial-derived products such as LPS (a TLR4 ligand) can induce a systemic inflammation, producing a more severe neurodegeneration. Extracellular α-synuclein is recognized as a DAMP and TLR4 ablation has been shown to impair the phagocytic response of microglia to α-synuclein with consequent accumulation of α-synuclein and increased dopaminergic neurodegeneration in the SNc [92], highlighting the neuroprotective effect of TLR4 signaling mediated through the clearance of α-synuclein, analogously to the suggested protective role of TLR2 towards β-amyloid and α-synuclein.

It has been demonstrated that bacteria residing in the enteric microbiota produce extracellular amyloid proteins. Curli, an amyloid protein synthesized by *E. coli* and *S. typhimurium*, has been shown to enhance colonization and biofilm formation. Even if few studies have focused on the presence of gut bacteria-derived amyloid proteins in the GI tract, the effect of bacterial amyloids on α-synuclein accumulation or other molecular mechanisms associated to neurodegeneration is still unknown [93]. The enteric microbiota is a huge antigenic load resident in the gut and confers marked potential danger if not kept under continuous surveillance, such as under TLRs sensing. However, the enteric commensal microbiota is required for the constant stimulation of the immune system and TLR-mediated sensing of these microorganisms may play a dual role in disease development as a source of both inflammatory and regulatory signals. In this respect, it is important to take into account that the microbiota is also a source of biological active signaling molecules, immune mediators and gut hormones. Some of those, including serotonin, purines, GABA and neurotrophic factors, among others, have been shown to be involved in TLRs signaling [72,73,94,95,96,97]. Recent evidence has also pointed out that microbiome-derived SCFAs finely tune microglial function, further suggesting that microbiome-derived factors and nutrition are involved in controlling innate immune function in neurodegeneration [98]. Intriguingly, the SCFA butyrate has been shown to increase TLR-dependent responses by increasing their expression in a cellular model of human enteroendocrine L-cells [99].

The emerging evidence described above suggests the existence of a multifaceted TLR signaling network that influences neural circuits and immune-mediated processes both in the gut and in the brain. Further studies focused on discovering the enteric microbiota-derived factors responsible of TLRs engagement and the consequent signaling outcomes of TLR activation in both the ENS and the CNS will provide novel insights into the complex dialogue between the host and the microbiota in PD and other relevant neurodegenerative disorders.

## 8. Potential Therapeutic Strategies

Recent research has gathered solid scientific evidence on the involvement of the GI tract in PD, highlighting three important features. First, in animal models and human studies a clear association has been put forward between the GI tract, gut inflammation, increased permeability, and PD. Second, some dietary interventions appear to exert beneficial effects by modulating microbiota dysbiosis toward a healthy state, reducing gut permeability, and/or decreasing oxidative stress and intestinal inflammation. Third, since TLRs are dynamic guardians laying squarely between the environment and the host, especially in the gut, any changes in TLR activity and expression might reduce or prevent the incidence of developing diseases associated with an inflammatory status such as PD. Deciphering the interactive dialogue that occurs between microorganisms in the gut and the activity of the immune system regulated by TLRs is crucial for the discovery and development of compounds such as pharma- and nutraceuticals (including syn-, pre- and probiotics), critical for preserving or restoring homeostasis in the gut as well as in other host tissues.

None of the current medicines for PD show a beneficial influence on disease progression and do not exert any effects on the microbiota-gut-brain axis to reduce the spread of Lewy pathology or to alleviate motor and/or non-motor symptoms. However, traditional treatments for PD could be combined with modulators of TLRs activity and/or food-based interventions to alleviate gut dysfunction and symptoms, as well as positively influence the microbiota-gut-brain axis, thus reducing GI dysbiosis and protecting the complex neuroglia network in both the ENS and CNS, [41,100,101,102,103].

### 8.1. Dietary Supplements

High levels of polyunsaturated fatty acids (PUFA), such as the omega-3 fatty acid docosahexaenoic (DHA), have been shown to induce anti-inflammatory effects and reduce mitochondrial dysfunction-mediated motor symptoms together with decreasing alpha-synuclein accumulation and inflammation in PD animal models [102,104,105,106,107]. DHA inhibits, whereas saturated fatty acids can activate, certain TLR-mediated pro-inflammatory signaling pathways. DHA blocks the activation of TLR4 and TLR2/1 or TLR2/6 and other TLRs in an indirect manner, targeting TLRs downstream pathways during the receptor dimerization process (e.g., lipid rafts) [108]. Overall these findings highlight the involvement of diet, such as the intake of saturated fatty acids and DHA, in the modulation of TLR signaling pathways and related involvement in chronic inflammation and subsequent risk of chronic diseases [108,109]. An in-vitro study has shown that an extract of *Panax notoginseng* was able to suppress microglial activation and decrease the release of inflammatory factors (IL-6 and TNF-α), suggesting the potential therapeutic utility in slowing down PD progression [110]. The flavonoid silymarin, extracted from the seeds and fruit of *Silybum marianum*, was found to exert neuroprotective effects in 6-OHDA-induced hemi-parkinsonian rats, through the alleviation of nigral injury, the increase of anti-oxidant defenses and suppression of TLR4 activation [111].

### 8.2. Probiotics

Probiotics are defined by the Food and Agriculture Organization of the United Nations and by the World Health Organization (FAO/WHO) as “live microorganisms which, when administered in adequate amounts, confer a health benefit on the host” [112]. The common benefit of probiotics on gut microbiota originates from creating a more favorable GI environment and supporting a healthy immune system [112]. Common probiotic functions are dependent on key core mechanisms found in the shared architecture of the cell surface structures of most Gram-positive probiotics, such as peptidoglycan, cell wall teichoic and lipoteichoic acid (LTA), and common but varying components including exopolysaccharides, surface layer associated proteins (SLAPS), mucin-binding proteins (MUBs), fibronectin binding proteins, and pili. These cell surface macromolecules in bacteria are important factors in the beneficial microbiota-host dialogue, as they can interact directly with the intestinal epithelium, mucus, and TLRs of the GI mucosa. The traditionally-used probiotics are from the genera *Lactobacillus* and *Bifidobacterium*, which have been shown to enhance intestinal epithelial integrity, protect from gut barrier disruption, regulate mucosal immune system and inhibit pathogenic bacterial growth [112,113].

The most common probiotics belonging to the genera of *Lactobacillus* and *Bifidobacterium* are Gram-positive bacteria and present a well-conserved core molecular architecture of peptidoglycan and LTA, which are documented MAMPs interacting with TLR2/6 heterodimers. Modifications in LTA structure or removal of LTA have elicited significant anti-inflammatory consequences observed in mouse models of both colitis and colon cancer, suggesting that Gram-positive probiotics with decreased expression of LTA are more prone to modulate the anti-inflammatory immunological consequences in degenerative disorders [114].

The administration of probiotics has been shown to positively modulate brain function, including stabilizing anxiety-like [115] and depression-like behavior [116], through the microbiota-gut-brain axis [117]. Chronic administration of the *Lactobacillus* strain in healthy mice has been shown to reduce anxiety, depression and stress responses, and modulate the expression of central GABA receptors, but only when the vagus nerve function was maintained intact, thereby highlighting the critical role of autonomic transmission in the gut-brain axis [118]. Preclinical or clinical evidence on the beneficial effects of probiotics in PD are still very limited. Ingestion of a fermented milk, supplemented with multiple probiotic strains and prebiotic fiber, for four weeks was found to be superior to placebo (a pasteurized, fermented, fiber-free milk) in improving constipation in patients with PD [119]. Another study showed that fermented milk containing *Lactobacillus casei* strain Shirota for five weeks decreased abdominal pain and bloating, and improved stool consistency [120].

A recent well-designed double-blinded randomized clinical trial (RCT) by Ojetti [121] showed that *Lactobacillus reuteri* improved bowel movement frequency compared to placebo in patients with functional constipation.

As mentioned above, gut dysfunction, including constipation and gut dysbiosis, contributes to PD morbidity and participates in the pathological process of PD. Hence, the use of probiotics might offer relief from the complications as well as decrease gut permeability, microbial translocation and neuroinflammation in the ENS. Restoring gut function by administration of probiotics might lead to better levodopa adsorption and improve behavioral and cognitive performance, which are common symptoms in PD patients.

### 8.3. Prebiotics

Prebiotics are defined as “a selectively fermented ingredient that results in specific changes, in the composition and/or activity of the gut microbiota, thus conferring benefit(s) upon host health” [122]. Examples of prebiotics are inulin, galacto-oligisaccharides (GOS), fructo-oligosaccharides (FOS) and SCFAs. Considering that human enzymes are not able to digest complex carbohydrates, specific gut microbes are involved in fermenting carbohydrates to SCFAs (i.e., acetate, proprionate and butyrate), lactose, hydrogen, methane, and carbon dioxide, all metabolic products that, by lowering the pH of the intestinal milieu, inhibit the survival and proliferation of pathogenic bacteria [103]. Prebiotic fibers are well known for their beneficial effects on gut motility and immune function that are compromised in PD patients [123]. The chronic administration of GOS and FOS for five weeks increased the levels of brain-derived neurotrophic factor (BDNF) in the hippocampus of healthy rats, suggesting that prebiotics can be implicated in ensuring CNS neuroprotection [124]. Although several studies have shown the beneficial effect of prebiotics on GI function, immune response and neuroprotection, to date, no clinical study has evaluated their use in PD which could be highly interesting in view of the fact that PD patients have a lower abundance of SCFA-butyrate producing bacteria [61].

### 8.4. Antibiotics

Treating SIBO with antibiotics is a potential therapeutic strategy to improve the motor symptoms, reduce intestinal bacterial contents as well as gas production. However, the therapy for SIBO is challenging due to the absence of well-designed clinical trials in patients with or without PD and SIBO. Moreover, there is still an absence of consensus on the exact definition of SIBO together with specific diagnostic criteria that render the treatment complicated. Antibiotic therapy is usually a good option for overcoming SIBO; however, considering that SIBO is caused by Gram-positive and -negative anaerobes and aerobes, the choice of most appropriate antibiotic poses a challenge for gastroenterologists. Rifaximin, a broad-spectrum nonadsorbable antibiotic, might be effective in the treatment of SIBO for its action on Gram-negative and -positive aerobic and anaerobic bacteria [125]. While rifaximin is the only antibiotic FDA-approved for treatment of diarrhea-predominant IBS and the most studied antibiotic for the treatment of SIBO, no data on the use of rifaximin for treatment of SIBO in patients with PD is currently available. A variety of other antibiotics (e.g., metronidazole, ciprofloxacin, norfloxacin, amoxicillin-clavulanate, tetracycline, doxycycline and neomycin etc.) have been evaluated for curing SIBO but the lower quality and sample power of most RCT have yielded no clear consensus on the most appropriate type of antibiotic and related posology to be used. A growing body of evidence has suggested that minocycline elicits neuroprotective effects in PD, owing to its ability to restore gut microbiota balance (reduction of *Firmicutes*/*Bacteroidetes*). Clinically, Phase II trials have assessed its efficacy in PD patients and it is now under consideration for undertaking a Phase III trial [126].

### 8.5. TLRs Modulators

As mentioned above, TLRs appear to be associated with the neurodegenerative processes characteristic of PD. TLR2 and TLR4 are potentially the most involved receptors in the progression of PD. However, it was not clear until now whether the most beneficial outcome would be achieved by stimulating TLR2 and/or TLR4 (e.g., favoring alpha-syn clearance) or to repress their signaling to delimitate Lewy pathology as well as augment the levels of toxic factors such as proinflammatory cytokines and reactive oxygen and nitrogen species. Considering that chronic administration of TLR2 and TLR4 ligands elicits microglial activation and a related marked release of toxic factors, current knowledge in other pathologies advocates for their inhibition to prevent further tissue damage. Several negative regulatory mechanisms have been developed to mitigate TLR signaling and ensure a balanced immune response. To date, six levels of negative regulation have been revealed: (i) degradation of TLR protein; (ii) down regulation of transcription mechanisms of TLR and related genes; (iii) post transcriptional repression by microRNAs (miRNAs); (iv) release of soluble TLRs acting as soluble decoy; (v) intracellular inhibitors; and (vi) membrane-bound blockers of TLR signaling pathways after TLR engagement by its ligands [127]. Preclinical and clinical studies have evaluated Eritoran tetrasodium, a compound that interferes with TLR4/MD-2/LPS, inhibiting the LPS-induced release of TNF-α, IL-β and IL-6, thus limiting excessive inflammation associated with TLR4 activation, as a potential therapeutic for sepsis. However, as far as we know, the efficacy of Eritoran has never been tested in PD [128]. Ibudilast is a cyclic nucleotide phosphodiesterase inhibitor as well as a TLR4 antagonist which is currently used as a bronchodilator, vasodilator and anti-inflammatory agent for the treatment of asthma, post-stroke dizziness and ocular allergies in Japan and other Asian countries [129]. Considering its documented ability on up-regulating the anti-inflammatory cytokines (i.e., IL-10, IL-4) and promoting the production of neurotrophic factors (i.e., GDNF, NGF, NT-4), it has been recently tested in an animal model of PD. Ibudilast showed an anti-inflammatory response via the modulation of glial cell activity, attenuation of TNF-α expression and induction of GDNF. However, these ibudilast-mediated beneficial events were not a sufficient protection in the acute phase of injury [129]. Anti-TLR2 antibodies have been tested in clinical studies for treating severe sepsis and inflammation. It is conceivable that these TLRs signaling inhibitors could modulate the progression of the severity of PD.

MicroRNAs (miRNAs) are recognized to be involved in the control of disease progression. Very recently it has been shown that the overexpression of miR-22 was induced by microbiota-produced butyrate [130]. The study of gut microbiota–miRNA interplay has disclosed that the gut microbiota may affect the host by producing miRNAs, and on the other side the gut microbiota might be regulated by host-secreted miRNAs [131]. An interesting study has highlighted that gut microbiota modulates miRNA-associated mRNA expression patterns in the hippocampus of germ-free mice. In addition, these transcriptional changes are sex-dependent, pointing towards a divergence between molecular pathways that control the gut-brain axis [132]. Some new findings advocate that miRNAs control the TLR-signaling pathway at several stages, including the regulation of TLR mRNA expression, direct activation of the receptor, binding to TLR or TLR-specific signaling pathway components and TLR-induced transcription factors and functional cytokines [133]. The research of the possible relationships between exosomes, miRNAs and TLRs in the nervous systems is still in its infancy. However, we can hypothesize that miRNAs entering the cells via exosomes may finely tune the activation of TLRs. Furthermore, TLR tolerance, a hyporesponsive state of the receptor, characterized by reprogramming of TLR-mediated signal transduction, may be achieved by intracellular delivery of miRNA using exosomes.

Potential pharma- and/or nutraceutical modulators of the microbiota-gut-brain axis as well as TLRs signaling in the progression of pathology in PD are summarized in Table 1.

## 9. Conclusions

Currently, no treatment for curing PD is available. Levodopa is the primary anti-parkinsonian medicine, which exerts a symptomatic effect but does not stop neurodegeneration and is ineffective on non-motor dysfunctions. Therefore a better understanding of the interaction between TLRs and the enteric microbiota-gut-brain axis might help to generate novel insights into PD pathology, as well as lead to new therapeutic strategies, such as pharmacological or dietary approaches. There is now mounting evidence for the beneficial effects of probiotics on ameliorating intestinal epithelial barrier function, stimulating host homeostasis of the mucosal immune system and preventing pathogenic microbial growth and colonization. Considering that TLR ligands derived from probiotics could suppress inflammation partially through the production of anti-inflammatory cytokines, the use of probiotics, or prebiotics or synbiotics, appears to be an interesting strategy, given their huge potential as medications or prophylactic agents against neurodegeneration. This potential stems from the fact that they exert a beneficial effect on the composition and function of the gut microbiota, restoring the complex dialogue between enteric microbes and the host, and ultimately reestablishing a balanced gut-brain axis.

## Figures and Tables

**Table 1:** Pharma- and/or nutraceuticals as potential modulators of the microbiota-gut-brain axis and Toll-like receptors (TLRs) signaling in the progression of pathology in Parkinson’s disease (PD).

Modulator	Influence on Microbiota-Gut-Brain Axis	Potential TLRs Target	References
*Dietary supplements*			
Docosahexaenoic acid (DHA)	Reduction of oxidative stress by improving neuronal mitochondrial dysfunction	TLR2, TLR4	[104,108,134]
*Panax Notoginseng* (*NotoG*)	Suppression of microglial activation and reduction of IL-6 and TNF-α release	TLR4	[110]
Sylimarin	Antioxidant and neuroprotective effects (salvaging of free radicals)	TLR4	[111]
*Probiotics*			
*L. rhamnosus* (*JB-1*)	Modulation of GABA_A_ and GABA_B_ receptors in the brain	TLR1, TLR2, TLR6	[118,135]
*Lactobacillus casei Shirota*	Decrease of visceral pain and bloating; improvement of stool consistency	TLR1, TLR2, TLR6	[120,135]
*Lactobacillus reuteri*	Improvement of bowel movement and increase of the frequency of evacuation	TLR1, TLR2, TLR6	[121,135]
*Prebiotics*			
Fructo oligosaccharides (FOS)	Increase of BDNF expression in the hippocampus	TLR2, TLR4	[73,124,135]
Galacto oligosaccharides (GOS)	Improvement of villus surface area in the small intestine	TLR1-TLR13	[135,136]
Short-chain fatty acids (SCFA)-Butyrate	Maintenance of colonic epithelium integrity	TLR1, TLR2, TLR6	[135,137]
*Antibiotics*			
Rifaximin	Treatment of small intestinal overgrowth	TLR4	[125]
Mynocicline	Neuroprotective effect on nigrostriatal dopaminergic neurons	TLR4	[126]
*TLRs modulators*			
Eritoran tetrasodium	Inhibition of LPS-induced proinflammatory cytokine release	TLR4	[128]
Ibudilast	Improvement of anti-inflammatory cytokine release, modulation of glial cells activity and induction of GDNF expression	TLR4	[129]
MiR-22	Induced by butyrate-producing commensal bacteria	TLR1, TLR2, TLR6	[130,135]
